# Therapeutic drug monitoring of colistin in patients with carbapenem-resistant infections and varying renal function: a series case report of 5 patients

**DOI:** 10.1097/MD.0000000000050278

**Published:** 2026-08-21

**Authors:** Qiuyu Zhu, Juan Wang, Fangqiong Li, Ji Shen, Xiaoting Gong, Bo Yang, Xufen Xia, Yuexing Tu

**Affiliations:** a School of Medical Technology and Information Engineering, Zhejiang Chinese Medical University, Hangzhou, Zhejiang, China; b Department of Clinical Laboratory, Tongde Hospital of Zhejiang Province Affiliated to Zhejiang Chinese Medical University (College of Integrated Traditional Chinese and Western Medicine Clinical Medicine), Hangzhou, Zhejiang, China; c Department of Pharmacy, Tongde Hospital of Zhejiang Province Affiliated to Zhejiang Chinese Medical University (College of Integrated Traditional Chinese and Western Medicine Clinical Medicine), Hangzhou, Zhejiang, China; d Department of Critical Care Medicine, Tongde Hospital of Zhejiang Province Affiliated to Zhejiang Chinese Medical University (College of Integrated Traditional Chinese and Western Medicine Clinical Medicine), Hangzhou, Zhejiang, China.

**Keywords:** colistin, liquid chromatography–triple quadrupole mass spectrometry, plasma concentration, renal function, therapeutic drug monitoring

## Abstract

**Rationale:**

Carbapenem-resistant gram-negative bacterial infections remain a challenge. Colistin is an important treatment option but exhibits substantial pharmacokinetic variability and nephrotoxicity, particularly in critically ill patients with altered renal function.

**Patient concerns:**

Five critically ill patients (aged from 25 to 94) with infections in the setting of heterogeneous underlying conditions, including hematologic malignancy, acute respiratory failure, severe trauma, chronic obstructive pulmonary disease, and acute cerebral infarction. Their renal function varied markedly, ranging from augmented renal clearance to severe renal impairment.

**Diagnoses:**

Carbapenem-resistant *Klebsiella pneumoniae*, carbapenem-resistant *Acinetobacter baumannii*, or both were identified during hospitalization. The patients exhibited different renal function profiles. Acute kidney injury was observed in cases 2 to 5, and cases 3 to 5 required continuous renal replacement therapy.

**Interventions:**

All patients received intravenous colistin sulfomethate sodium, with plasma colistin concentrations quantified by liquid chromatography–triple quadrupole mass spectrometry; dosing was adjusted according to clinical course and renal function.

**Outcomes:**

Colistin plasma concentrations varied considerably among patients and were not simply inversely related to renal function. All patients achieved the ratio of the unbound area under the concentration–time curve to the minimum inhibitory concentration target for systemic infection, and 3 additionally achieved the higher target for pulmonary infection; however, a favorable clinical outcome was observed in only 2 of the 5 cases.

**Lessons:**

Colistin exposure cannot be predicted only from renal function; therapeutic drug monitoring may assist in individualized colistin sulfomethate sodium dosing, particularly in patients with augmented renal clearance or markedly altered renal function. Moreover, the target of unbound area under the concentration–time curve to the minimum inhibitory concentration attainment does not necessarily translate into clinical success, indicating that clinical efficacy is also influenced by disease severity and patient-specific factors.

## 1. Introduction

Over the past decades, inappropriate antibiotic use has led to a considerable increase in the number of multidrug-resistant and extensively drug-resistant gram-negative strains, such as those of *Pseudomonas aeruginosa*, *Acinetobacter baumannii*, and Enterobacteriaceae species. These strains cause infections associated with high morbidity and mortality, making these organisms an urgent global public health issue.^[1–4]^ However, effective antibiotic treatments for these infections remain unavailable. Delays in the release of newer antimicrobial agents targeting carbapenem-resistant gram-negative bacilli (CR-GNBs) have further limited the treatment options.^[5]^ In current clinical practice, colistin, an older drug, is used as the last resort to mitigate the worsening resistance trends.

Colistin, also known as polymyxin E, is a cationic polypeptide antibiotic produced by *Paenibacillus polymyxa* subsp. *colistinus*. It demonstrates potent anti-CR-GNB activity by binding to the negatively charged sulfate portion of lipid A in the lipopolysaccharides of the organisms’ outer cell membranes. The interactions between colistin and CR-GNBs result in outer membrane disorganization, followed by intracellular content leakage and bacterial death.^[6,7]^ Colistin sulfomethate sodium (CMS), formed by the reaction of sulfomethyl groups with the primary amines of colistin, is an inactive prodrug. Thus, it can be hydrolyzed and converted to colistin, which exerts antibacterial effects.^[8]^ However, CMS induces various adverse reactions, primarily nephrotoxicity and neurotoxicity. This is one of the main reasons colistin was contraindicated in clinical practice during the 1970s. The historical withdrawal of CMS from clinical use prevented it from undergoing contemporary drug development and regulatory approval processes, leading to significant deficiencies in its pharmacokinetics/pharmacodynamics (PK/PD) data. Thus, little is known about the optimal strategies for CMS dosing, especially in specific populations, including critically ill, obese, and renally impaired patients.^[9,10]^

Therapeutic drug monitoring (TDM) originated in the 1950s and is an essential tool, integrating modern analytical techniques and pharmacology to help clinicians understand metabolism, bioavailability, toxicity, and pharmacokinetics, thereby facilitating prompt dose individualization to maximize therapeutic outcomes.^[11,12]^ Currently, various medications, such as antiepileptic drugs, immunosuppressive drugs, antibiotics, antifungal drugs, cardiovascular agents, and anticancer drugs, are monitored using clinical TDM.^[11]^ However, to the best of our knowledge, TDM has rarely been used for colistin in clinical practice. Colistin demonstrates a narrow therapeutic window, high toxicity,^[13,14]^ and a wide plasma concentration range.^[10]^ Therefore, reliable TDM is imperative to ensure that each patient receives colistin at concentrations within the therapeutic range.^[15]^

Accurate analytical methods are necessary for TDM. Over the past decades, multiple methods have been established to measure colistin levels in biological samples. Liquid chromatography-tandem mass spectrometry has gained clinical prominence owing to its enhanced sensitivity, superior selectivity, and rapid analysis.^[16]^ The liquid chromatography–triple quadrupole mass spectrometry (LC-TQ-MS) method we employ is also a subset of liquid chromatography-tandem mass spectrometry. Due to the complexity of biological samples containing abundant endogenous interferents, pretreatment is a critical step in the analytical procedure. In recent years, magnetic solid-phase extraction (MSPE), characterized by operational simplicity, rapid separation, exceptional adsorption efficiency, and eco-compatibility, has emerged as a novel pretreatment method. It uses an external magnetic field to capture target analytes, eliminating the need for centrifugation or filtration.^[17,18]^ It has been employed in several pharmaceutical and biomedical analyses, such as antitumor agents,^[19]^ hypoglycemic drugs,^[20]^ and anesthetics.^[21]^ However, its application in the determination of plasma colistin concentration has not been reported.

Herein, we report 5 cases of carbapenem-resistant *Klebsiella pneumoniae* (CRKP) and carbapenem-resistant *A baumannii* (CRAB) infections in patients with varying renal function, all of whom were treated with CMS. In each case, we evaluated efficacy and safety by integrating plasma colistin concentrations (measured via LC-TQ-MS combined with MSPE) with relevant clinical parameters.

## 2. Methods

### 2.1. Case design and data collection

This retrospective study included critically ill patients who received intravenous CMS (brand name Tiance; Chia Tai Tianqing Pharmaceutical Group, Nanjing, China) treatment for CRKP or CRAB infection. Patients were administered CMS for at least 48 hours. All dosages were uniformly expressed in million international units (MIU) following the standard conversion (1 million IU ≈ 30 mg colistin base activity). The plasma colistin concentration reached a steady state, which was typically achieved by the fourth or fifth dose.^[22]^

The following data were collected retrospectively from medical records: demographic characteristics, including age, sex, body weight, hospital stay length, and admission and discharge dates; inflammatory markers, such as the highest temperature at hospitalization, white blood cell (WBC) count, C-reactive protein (CRP) level, and procalcitonin (PCT) level; clinical information, such as diagnosis at admission and comorbidities; TDM results, such as maximum steady-state concentration and minimum steady-state concentration. *C*_min_ and *C*_max_ were measured 30 minutes before and after the fourth or fifth dose, respectively; and information regarding CMS treatment, including the prescribed regimen, concomitant nephrotoxins, and whether a loading dose was administered.

### 2.2. Definition

#### 2.2.1. Outcomes

The primary outcomes were clinical and microbiological responses. A favorable clinical response was defined as the disappearance of infection symptoms and decreases in inflammatory marker levels. In contrast, persistence or worsening of the patient’s condition, or even death, during hospitalization was considered to indicate clinical failure. A favorable microbiological response was defined as the eradication of CRKP or CRAB at the end of CMS treatment. However, the persistence of CRKP or CRAB at the end of CMS treatment suggested ineffective therapy.

Nephrotoxicity during CMS treatment was regarded as the secondary outcome. Acute kidney injury (AKI) was defined according to the Kidney Disease: Improving Global Outcomes (KDIGO) creatinine criteria^[23]^: an increase in serum creatinine (Scr) by ≥0.3 mg/dL (≥26.5 µmol/L) within 48 hours or an increase in Scr to ≥1.5 times the baseline, which is known or presumed to have occurred within the prior 7 days. AKI was staged as follows^[23]^: Stage 1 was considered in cases of a Scr increase of 1.5 to 1.9 times from baseline, or an absolute increase of ≥0.3 mg/dL (≥26.5 μmol/L). Stage 2 was considered in cases of a Scr increase of 2.0 to 2.9 times from baseline. Stage 3 was considered in cases of a Scr increase of ≥3.0 times from baseline, an increase to a level of ≥4.0 mg/dL (≥353.6 μmol/L), the initiation of renal replacement therapy, or, in patients under 18 years of age, a decrease in eGFR to <35 mL/min/1.73 m^2^

#### 2.2.2. Renal function

The following criteria were applied to stratify renal function based on creatinine clearance (CrCl) values: augmented renal clearance (ARC; ≥130 mL/min/1.73 m^2^),^[24]^ normal (≥90 mL/min/1.73 m^2^), mild impairment (60–89 mL/min/1.73 m^2^), moderate impairment (30–59 mL/min/1.73 m^2^), and severe impairment (15–29 mL/min/1.73 m^2^).^[25]^ Scr levels were recorded from the day of admission until discharge or death. CrCl was calculated using the Cockcroft-Gault formula:


$$CrCl\left(mL/min\right)=\frac{\left(140-Age\right)\times Actual\;body\;weight\;\left(kg\right)}{72\times Serum\;creatinine\;\left(mg/dL\right)}$$

Baseline renal function was evaluated at admission. Subsequent changes in renal function were monitored throughout the therapy.

#### 2.2.3. Microbiology

The minimum inhibitory concentrations (MICs) of colistin were determined using the broth microdilution method, following the Clinical and Laboratory Standards Institute guidelines as the reference standard. Fresh solutions of colistin sulfate were prepared and serially diluted in cation-adjusted Mueller-Hinton broth in standard polystyrene plates. Quality control was performed using *Escherichia coli* ATCC 25922 and *P aeruginosa* ATCC 27853 to ensure accuracy.

#### 2.2.4. Pharmacokinetic/pharmacodynamic index and efficacy targets

The antibacterial activity of colistin is concentration-dependent. The ratio of the area under the unbound drug concentration-time curve to the MIC (*f*AUC/MIC) has been identified as the PK/PD index most strongly correlated with its antibacterial effect.^[8]^ We employed *f*AUC/MIC as the primary index for evaluating colistin efficacy. For systemic infections, an *f*AUC/MIC value of ≥12 was set as the efficacy target, as this corresponds to an approximate 2-log10 bacterial reduction. For pulmonary infections, which demonstrate reduced responsiveness, a higher target of *f*AUC/MIC of ≥48 was applied.^[26,27]^

To calculate the individual *f*AUC/MIC ratio for each patient, the following steps were undertaken. The AUC_ss,24h_ (24-hour steady-state area under the concentration-time curve) was derived from the TDM data. The LC-TQ-MS method used in our study quantified the total plasma concentration of colistin (bound plus unbound). Recognizing that colistin is approximately 50% unbound in human plasma (unbound fraction, *f*u = 0.5),^[26]^ the unbound AUC (*f*AUC) was calculated by multiplying the total AUC_ss,24h_ by the unbound fraction: *f*AUC = AUC_ss,24h_ × 0.5. The resultant *f*AUC value was then divided by the MIC of the causative pathogen (e.g., CRAB or CRKP) for the respective patient.

### 2.3. Determination of colistin plasma concentration by LC-TQ-MS

The concentrations of colistin A and B were measured individually, and their sum was used to calculate the total colistin content. Polymyxin B1 (Aladdin Biochemical Technology Co., Ltd., Shanghai, China) was used as the internal standard (IS). The analytical method was validated for key parameters, including specificity, linearity, precision, accuracy, recovery, matrix effect, and stability, in accordance with the Bioanalytical Method Validation of the International Council for Harmonisation of Technical Requirements for Pharmaceuticals for Human Use (ICH M10 guideline). The detailed validation procedures were implemented as described in our separate work.^[28]^ Excellent linearity was observed for both colistin A (*y* = 9.55*x* + 0.0842, *r* = 0.9994) and colistin B (*y* = 8.21*x* − 0.0036, *r* = 0.9997) across the 0.01 to 20 µg/mL range. For colistin A, intra- and inter-day accuracy were −7.0% to 8.2% and −6.9% to 3.1%, with relative standard deviation (RSD) of 2.9% to 9.5% and 3.1% to 8.1%, respectively. For colistin B, the corresponding accuracy values were −6.9% to 8.5% and −9.5% to 3.3%, with RSDs of 3.0% to 7.3% and 2.8% to 9.2%. The matrix effect for colistin A and B was minimal, ranging from 97.4% to 107.2%, with RSD of ≤3.7% across different matrix sources, indicating no significant ionization suppression or enhancement. The absolute extraction recovery was consistent, being 81.4% to 87.6% for colistin A, 81.2% to 85.2% for colistin B, and 80.6% to 87.8% for the IS. Consequently, the IS-normalized recovery demonstrated excellent efficiency, ranging from 97.9% to 101.3% with an RSD of ≤8.7%. Carryover was shown to be negligible, with blank samples injected after the highest calibrator (20 µg/mL) producing analyte responses below 20% of the lower limit of quantification and IS responses under 5% of the average peak area. Colistin A and B were stable in whole blood (room temperature, ≤6 hours), through 3 freeze-thaw cycles, and in processed samples (autosampler at 4°C for 24 hours). Storage stability was also confirmed under short-term conditions (room temperature, 4°C, and −20°C for up to 24 hours; −20°C for 21 days) and long-term conditions (−80°C for 21 days).

## 3. Case descriptions

### 3.1. Case 1

A 25-year-old man diagnosed with acute myeloid leukemia, sepsis, bone marrow suppression after chemotherapy, and pulmonary infection was transferred to the hematology department. At admission, he had a high fever (39.9°C), high CrCl (149.56 mL/min/1.73 m^2^), and high inflammatory marker levels (WBC: 16.5 × 10^9^/L, CRP: 21.3 mg/L, PCT: 2.005 ng/mL). Pulmonary computed tomography (CT) revealed patchy shadows in the upper lobes of both lungs. Because he was suspected to have multiple bacterial infections involving *E coli*, *Enterococcus faecium*, *Mycobacterium tuberculosis* complex, and *A baumannii*, the initial treatment included ceftazidime-avibactam, along with isoniazid, linezolid, isavuconazole, and amphotericin B cholesterol sulfate. During this period, he had a low-grade fever and gastrointestinal bleeding. On day 7, follow-up pulmonary CT demonstrated partial absorption of the original lesion in the right upper lobe with some new inflammation. Thus, on day 15, the antibiotic regimen was changed to ceftazidime-avibactam alone; however, his body temperature remained high and fluctuating. On day 21, next-generation sequencing revealed positivity for metallo-β-lactamase-producing *K pneumoniae*, *Enterobacter cloacae,* and cytomegalovirus (CMV); moreover, his inflammatory marker levels significantly increased (WBC: 10 × 10^9^/L, CRP: 26 mg/L, PCT: 2.985 ng/mL), and his body temperature increased to 40.5°C. A combination of tigecycline (TGC) and CMS (4.5 MIU q12h), along with isoniazid, was administered for infection. On day 24, TDM was implemented. Blood culture remained positive for CRKP, and his temperature fluctuated between 38°C and 40°C. Because of the unsatisfactory treatment outcome, the patient requested discharge on day 27.

### 3.2. Case 2

A 60-year-old man was admitted to the EICU for abdominal pain accompanied by cough and expectoration for 8 days and fever for 1 day. He was diagnosed with a pulmonary infection, acute respiratory failure, intestinal obstruction, and heart failure. At admission, he had elevated inflammatory marker levels (WBC: 13.8 × 10^9^/L, CRP: 147.2 mg/L, PCT: 2.027 ng/mL). His chest CT showed bilateral pleural effusion. His body temperature was 38.1°C, and his CrCl was 98.28 mL/min/1.73 m^2^, indicating normal renal function. Initially, ertapenem (ERT) combined with meropenem (MEM) was administered to treat the infection. Under this treatment plan, CRP levels continued to decline. However, on day 6, sputum culture revealed CRAB, and the inflammatory marker levels began to increase again. Therefore, antibiotic therapy was changed to intravenous cefoperazone sodium/sulbactam sodium (CPZ/SBT) and CMS (4.5 MIU q12h), supplemented with nebulized CMS (2.25 MIU q12h). On day 12, the sputum culture was negative for CRAB but positive for CRKP. CPZ/SBT was replaced with intravenous TGC to target CRKP, and TDM was implemented. Under this regimen, the patient’s CRP levels demonstrated a downward trend. On day 17, his condition improved, and CRKP were cleared, and the antibiotics were discontinued. The patient developed KDIGO stage 1 AKI, and his CrCl decreased to 41.45 mL/min/1.73 m^2^.

### 3.3. Case 3

A 47-year-old man who fell from a height was admitted to the EICU. He had sustained severe craniocerebral injury, pulmonary infection, and acute respiratory failure. At admission, he had a normal body temperature (36.7°C) and a CrCl of 60.1 mL/min/1.73 m^2^. On day 2, he underwent an epidural hematoma removal procedure. After surgery, he developed a high fever (38.8°C) and elevated inflammatory marker levels (WBC: 20.9 × 10^9^/L, CRP: 76.2 mg/L, PCT: 12.843 ng/mL). Blood culture suggested a *Staphylococcus aureus* infection. Thus, piperacillin-tazobactam (PIP-TAZ) was administered. On day 4, the sputum culture was negative for *S aureus* but positive for CRAB. Therefore, the antibiotic was changed to MEM combined with methylprednisolone. Over the next few days, his inflammatory marker levels and body temperature remained high. On day 10, the regimen was changed to CPZ/SBT along with TGC. On day 14, the infection worsened, with the sputum culture remaining positive for CRAB, which was sensitive to CMS (MIC ≤ 0.5) only. His CrCl was 50.2 mL/min/1.73 m^2^, indicating moderate renal function impairment. Thus, CPZ/SBT was replaced with CMS (2.25 MIU q12h) to target CRAB. On the same day, he developed KDIGO stage 1 AKI. On day 16, his condition progressed to sepsis, complicated by septic shock and AKI (KDIGO stage 2), with persistently high inflammatory marker levels (WBC: 16.2 × 10^9^/L, CRP: 224.8 mg/L, PCT: 11.436 ng/mL). Thus, continuous venovenous hemofiltration (CVVH) was performed using a Prismaflex ST150 system (Gambro AB, Lund, Sweden) and an AN69 ST membrane (surface-treated acrylonitrile-sodium-methyl sulfonate; 1.5 m^2^ surface area). The treatment parameters were set as follows: blood flow rate (BFR) 150 mL/min, a balanced pre-dilution and post-dilution regimen (1000 mL/h each), net ultrafiltration rate (UFR) 100 mL/h, and sodium bicarbonate supplementation at 150 mL/h, conducted without heparin anticoagulation. The total effluent rate was 2250 mL/h. The CMS dose was increased to 4.5 MIU (once every 12 hours). TDM was performed on the same day. After 4 days of CVVH, the mode was changed to continuous venovenous hemodiafiltration (CVVHDF). The CMS-based combination regimen was adjusted over the subsequent days, first to CPZ/SBT (on day 17) and then to eravacycline (on day 19). Even after 10 days of CMS treatment, digital polymerase chain reaction (PCR) remained positive for CRAB. Considering the severity of his condition, he was transferred to another hospital for further treatment.

### 3.4. Case 4

A 94-year-old man with prostate cancer, Alzheimer disease, and renal insufficiency was admitted to the ICU for acute exacerbation of chronic obstructive pulmonary disease and acute respiratory failure. At admission, he had a fever (38.6°C), low CrCl (25.49 mL/min/1.73 m^2^), and high inflammatory marker levels (WBC: 12.6 × 10^9^/L, CRP: 45.2 mg/L, PCT: 37.547 ng/mL). He was placed on mechanical ventilation and suspected of having aspiration pneumonia. On day 2, PCR indicated 224 copies/mL of *K pneumoniae*, suggesting a blood infection. He also demonstrated decreased urine output and gradually increasing Scr levels, resulting in KDIGO stage 1 AKI. Initially, MEM was administered. One day later, linezolid plus caspofungin were added. By day 6, PCR results became negative for *K pneumoniae*, indicating the effectiveness of the antibiotics. Thus, MEM was replaced with PIP-TAZ. On day 13, AKI progressed to KDIGO stage 3, and he was treated with pre-dilution CVVHDF using a Prismaflex ST150 system (Gambro AB, Lund, Sweden) equipped with an AN69 ST membrane (acrylonitrile-sodium-methyl sulfonate, 1.5 m^2^ surface area). Regional citrate anticoagulation was employed for circuit patency. The treatment parameters were as follows: a BFR of 150 mL/min, a pre-dilution replacement fluid rate of 1000 mL/h, and a dialysate flow rate of 1000 mL/h. The UFR was 200 mL/h. The filtration fraction was maintained below 25%. However, his renal function continued to deteriorate. On day 16, CRAB sensitive to colistin (MIC ≤ 0.5) was isolated from the sputum culture. His condition worsened with septic shock, persistent fever (37.4°C–38.5°C), and high inflammatory marker levels (WBC: 11.8 × 10^9^/L, CRP: 242.82 mg/L, PCT: 6.187 ng/mL). Therefore, the antibiotics were changed to TGC combined with CMS (2.25 MIU q12h). On day 19, TDM was performed during this regimen. On day 22, due to the continual deterioration of renal (CrCl: 8.76 mL/min/1.73 m^2^) and liver function, TGC was replaced with CPZ/SBT. After 9 days of CMS treatment, the sputum cultures turned negative for CRAB. Despite these interventions, the patient died of severe infection and multiorgan failure on day 24.

### 3.5. Case 5

A 72-year-old man was transferred to the ICU for acute cerebral infarction, pulmonary infection, and respiratory failure. He had previously received PIP-TAZ for the treatment of an infection and was on mechanical ventilation via endotracheal intubation. At admission, his temperature was 36.5°C, and the inflammatory markers were normal. He had chronic kidney disease stage 5 and KDIGO stage 3 AKI. On day 4, continuous renal replacement therapy was conducted using a Prismaflex ST150 system (Gambro AB, Lund, Sweden) equipped with an AN69 ST membrane (acrylonitrile-sodium-methyl sulfonate, 1.5 m^2^ surface area) in pre-dilution CVVHDF mode, anticoagulated with nafamostat mesilate. The BFR was initiated at 80 mL/min and gradually increased to 150 mL/min. The pre-dilution replacement fluid rate was set at 1000 mL/h, and the dialysate flow rate was set at 1000 mL/h. The UFR was 150 mL/h, with an actual ultrafiltration rate of 150 mL/h. The total effluent rate was calculated to be 2150 mL/h. Initially, PIP-TAZ was administered. On day 10, he developed a fever (37.8°C) with elevated inflammatory marker levels (WBC: 11.7 × 10^9^/L, CRP: 39.4 mg/L, PCT: 0.632 ng/mL). Subsequent sputum cultures indicated CRAB and CRKP infection; therefore, the antibiotic was adjusted to CPZ/SBT and CMS (2.25 MIU, q24h) intravenously, plus CMS (2.25 MIU q12h) via nebulization inhalation. To optimize the CMS dosing, TDM was implemented on day 13. On day 15, his clinical infection and inflammatory markers did not improve. The doses of CPZ/SBT and CMS were increased to 2 g once every 12 hours and 4.5 MIU once every 12 hours, respectively. Subsequently, his body temperature normalized, inflammatory marker levels decreased, and chest CT revealed improved pulmonary infiltrates. On day 20, sputum culture revealed clearance of CRAB and CRKP; however, it was positive for *Pseudomonas cepacia* and *Klebsiella oxytoca*, susceptible to MEM (MIC ≤ 0.25). Therefore, CMS was discontinued, and MEM was administered. On day 28, the patient was transferred to another hospital for further treatment.

## 4. Case summary

All the patients were male and aged 25 to 94 years. At admission, 3 patients (cases 1, 2, and 4) presented with fever and elevated levels of inflammatory markers. Cases 2 to 5 demonstrated acute respiratory failure and pulmonary infection. They were all infected with CRKP, CRAB, or both during a certain period of their hospitalization and were treated with intravenous CMS (Table 1).

**Table 1:** Demographic and clinical data of patients receiving CMS therapy.

Parameters	Case 1	Case 2	Case 3	Case 4	Case 5
Age (yr)	25	60	47	94	72
Sex	Male	Male	Male	Male	Male
Weight (kg)	70	60	80	60	60
Comorbidities	Acute myeloid leukemia; pulmonary infection; sepsis; post-chemotherapy bone marrow suppression	Pulmonary infection; acute respiratory failure; intestinal obstruction; heart failure	Craniocerebral injury; acute respiratory failure; pulmonary infection	COPD; Alzheimer disease; acute respiratory failure; prostate cancer	Acute cerebral infarction; pulmonary infection; acute respiratory failure; stage 5 CKD
Treatment regimens (d)	CAZ-AVI + INH + LZD + ISA + ABLC (7); CAZ-AVI (6); CMS + TGC (7)	ERT + MEM (5); CPZ/SBT + CMS (7); CMS + TGC (5)	PIP-TAZ (2); MEM (7); CPZ/SBT + CMS (3); CMS + TGC (3); CPZ/SBT + CMS (2); ERV + CMS (4)	MEM + LZD + CAS (4); PIP-TAZ + LZD + CAS (9); CMS + TGC (6); CPZ/SBT + CMS (3)	PIP-TAZ (6); CPZ/SBT + CMS (13); MEM (10)
CMS regimen	4.5 MIU q12h	4.5 MIU q12h	4.5 MIU q12h	2.25 MIU q12h	First 2.25 MIU q12h, then 4.5 MIU q12h
A loading dose	No	No	No	No	No
*C*_min,ss_ (mg/L)	1.464	1.833	3.213	2.667	2.282
*C*_max,ss_ (mg/L)	2.787	3.415	4.707	4.640	3.787
*C*_ss_,_avg_ (mg/L)	2.126	2.624	3.960	3.654	3.035
AUC_ss,24h_ (mg·h/L)	51.01	62.98	95.04	87.68	72.83
Organism/MIC	*K pneumoniae* (MIC = 2)	*K pneumoniae* (MIC = 2); *A baumannii* (MIC = 2)	*A baumannii* (MIC ≤ 0.5)	*K pneumoniae* (/); *A baumannii* (MIC ≤ 0.5)	*K pneumoniae* (MIC ≤ 0.5); *A baumannii* (MIC ≤ 0.5)
*f*AUC/MIC	12.75	15.75	95.04	87.68	72.83
Baseline renal function	ARC	Normal	Mild impairment	Severe impairment	Severe impairment
AKI stage	No	I	I, II	II, III	III
CRRT	No	No	CVVH + CVVHDF	CVVHDF	CVVHDF
Outcome	Ineffective	Effective	Ineffective	Ineffective	Effective

ABLC = amphotericin B cholesterol sulfate, AKI = acute kidney injury, AUC_ss,24h_ = 24-hour steady-state area under the concentration-time curve, CAS = caspofungin, CAZ-AVI = ceftazidime-avibactam, CKD = chronic kidney disease, *C*_max,ss_ = maximum steady-state concentration, *C*_min,ss_ = minimum steady-state concentration, CMS = colistin sulfomethate sodium, COPD = chronic obstructive pulmonary disease, CPZ/SBT = cefoperazone sodium/sulbactam sodium, CRRT = continuous renal replacement therapy, C_ss,avg_ = average steady-state plasma concentration, CVVH = continuous venovenous hemofiltration, CVVHDF = continuous venovenous hemodiafiltration, ERT = ertapenem, ERV = eravacycline, *f*AUC/MIC = the ratio of the area under the unbound drug concentration-time curve to the minimum inhibitory concentration, INH = isoniazid, ISA = isavuconazole, LZD = linezolid, MEM = meropenem, MIC = minimum inhibitory concentration, MIU = million international units, PIP-TAZ = piperacillin-tazobactam, TGC = tigecycline.

Four patients (cases 2–5) developed AKI. The timing of AKI onset varied: case 2 developed stage 1 AKI after 12 days of CMS treatment; case 3 developed AKI stage 1 on the first day of CMS treatment. His AKI progressed to stage 2 within 2 days. This progression occurred concurrently with a diagnosis of sepsis, which prompted the initiation of CVVH. After 5 days, the modality was switched to CVVHDF. For case 4, AKI stage 1 was noted on the second day of hospitalization. AKI stage 1 progressed to AKI stage 3 before CMS was administered, and CVVHDF was initiated. Case 5, who had preexisting chronic kidney disease stage 5, developed AKI stage 3 on the first day of hospitalization and commenced CVVHDF on day 4. The patient’s renal function improved after CVVHDF therapy. However, following the initiation of CMS treatment, AKI stage 3 recurred. Except for case 1, who received amphotericin B before CMS therapy, none of the other 4 patients (cases 2–4) were exposed to drugs with marked nephrotoxicity during the CMS treatment.

The PK parameters and PK/PD target attainment for all patients are summarized in Table 1. Case 1, who had ARC (CrCl 149.56 mL/min/1.73 m^2^), achieved an *f*AUC/MIC of only 12.75. Case 2, also infected with pathogens with a 2 mg/L MIC, achieved an *f*AUC/MIC of 15.75. While both values marginally surpassed the minimum target for general infections (*f*AUC/MIC ≥ 12), they were substantially below the recommended target of *f*AUC/MIC ≥ 48 for effective treatment of pulmonary infections. Notably, the corresponding microbiological outcomes diverged: case 1 exhibited a poor response with failure to clear CRKP, whereas case 2 achieved a favorable clinical and microbiological outcome, with successful eradication of both CRKP and CRAB. In contrast, all patients infected with more susceptible pathogens (MIC ≤ 0.5 mg/L) achieved robust colistin exposure. Cases 3, 4, and 5 attained *f*AUC/MIC values of 95.04, 87.68, and 72.83, respectively, comfortably exceeding both the general and pulmonary infection targets. Despite achieving substantial *f*AUC/MIC values, only case 5 demonstrated a favorable therapeutic response with microbiological clearance. The clinical outcomes in cases 3 and 4 were poor. Although microbiological clearance of CRAB was observed following CMS therapy in case 4, the patient ultimately succumbed. Case 3 was transferred to another hospital with a persistently positive CRAB status, indicating a failure of microbiological eradication.

## 5. Discussion

After administration, most CMS is excreted through glomerular filtration, tubular secretion, and certain nonrenal clearance pathways; therefore, only a small amount of the drug, which spontaneously converts into colistin, can exert antibiotic effects.^[29]^ Therefore, renal function status has a substantial impact on the plasma colistin concentrations,^[13]^ consequently affecting its efficacy and safety. In the present study, a validated MSPE-based LC-TQ-MS method was employed to determine plasma concentrations of colistin. The current TDM results confirmed that individual variations in renal function considerably affected colistin plasma concentrations among our patients.

**Figure 1.**
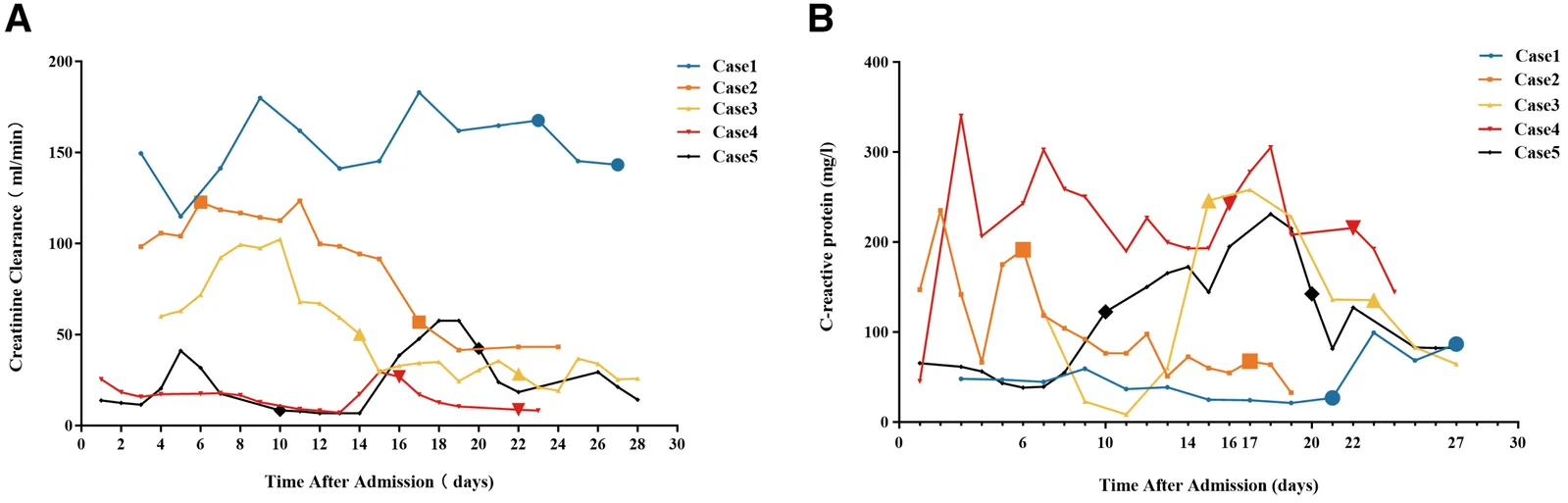
Trends of inflammatory marker and renal function indicator levels in all 5 cases during hospitalization. Changes in (A) CrCl and (B) CRP levels during hospitalization. Enlarged dots represent colistin administration periods. CrCl = creatinine clearance, CRP = C-reactive protein.

The dosing regimens of CMS are typically adjusted according to patients’ renal function, represented by CrCl, in actual clinical practice. In this study, during the CMS administration period, the CrCl of 4 cases (cases 1–4) decreased but with fluctuations (Fig. 1A), and the inflammatory marker CRP showed a generally similar trend (Fig. 1B). These observations confirmed the role of colistin in renal function impairment, as well as its effectiveness against CR-GNBs, corroborating previously reported results.^[30,31]^

**Figure 2.**
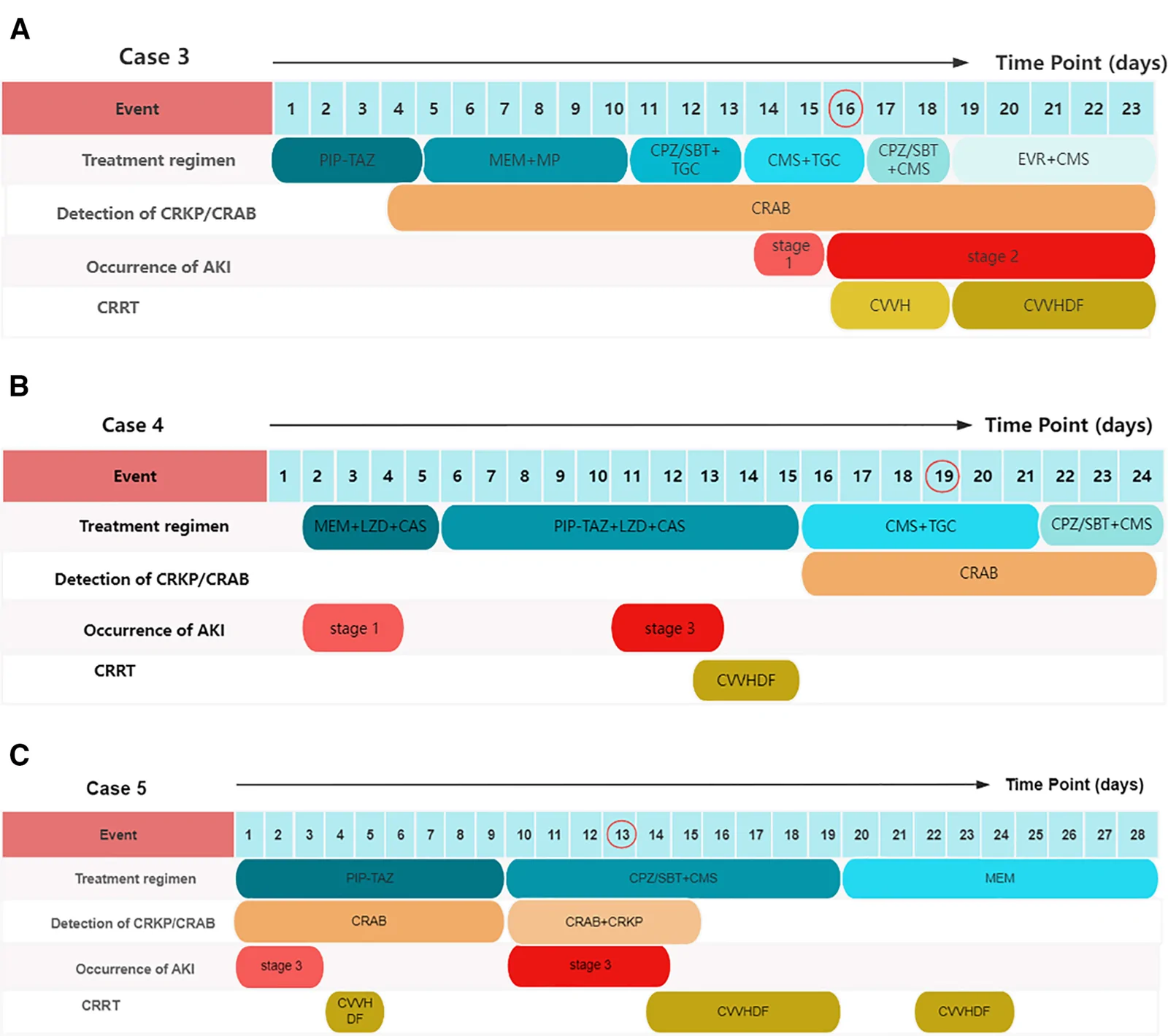
Clinical course of case 3 (A), case 4 (B), and case 5 (C) during hospitalization, including medication use, occurrence of acute kidney injury (AKI), requirement for continuous renal replacement therapy (CRRT), and detection of carbapenem-resistant *Acinetobacter baumannii* (CRAB) or carbapenem-resistant *Klebsiella pneumoniae* (CRKP). The red circles on the timeline represent the timing of TDM implementation (blood sample collection). CAS = caspofungin, CMS = colistin sulfomethate sodium, CPZ/SBT = cefoperazone sodium/sulbactam sodium, CVVH = continuous venovenous hemofiltration, CVVHDF = continuous venovenous hemodiafiltration, EVR = eravacycline, LZD = linezolid, MEM = meropenem, MP = methylprednisolone, TDM = therapeutic drug monitoring, PIP-TAZ = piperacillin-tazobactam, TGC = tigecycline.

Kim et al identified a negative correlation between CrCl and colistin plasma exposure: the more severe the renal insufficiency, the higher the colistin plasma concentration.^[32]^ Similarly, our study observed this trend. However, case 3 was an exception to the aforementioned phenomenon: although the patient had high colistin plasma concentrations, his renal function was only mildly impaired. Furthermore, he was receiving CVVH using an AN69 ST membrane when TDM was conducted (Fig. 2A). The AN69 ST membrane, a surface-treated acrylonitrile-sodium-methyl sulfonate copolymer, possesses a high density of negatively charged sulfonate groups. This enables the membrane to adsorb positively charged substances through electrostatic interactions.^[33]^ Being a polycationic lipopeptide, colistin is an excellent substrate for such electrostatic interactions. Consequently, a significant portion of the colistin can be sequestered onto the membrane surface and within the circuit tubing. This represents a non-convective, non-diffusive clearance mechanism that is not accounted for when calculating drug removal based solely on the effluent flow rate. Despite this potent adsorptive clearance, case 3 still developed higher colistin plasma concentrations than cases 4 and 5, who had severe renal impairment and, importantly, were not receiving continuous renal replacement therapy at the time of TDM (Fig. 2B and C). Thus, the colistin plasma concentration measured in cases 4 and 5 reflected their true systemic exposure, unaffected by extracorporeal clearance. It highlights the extreme interindividual variability in colistin PK among critically ill patients and reinforces the critical role of TDM for dose individualization.

In addition, cases 4 and 5 had severe renal impairment; both achieved the *f*AUC/MIC target for pulmonary and systemic infections. However, they demonstrated markedly different microbiological and clinical outcomes, which may be attributed to their advanced age. A recent multicenter cohort study by Huang et al provides compelling evidence for the profound impact of aging on outcomes in critical illness. Analyzing nearly 1995 acute respiratory distress syndrome patients, they found a significant association between increasing age and worse clinical endpoints. Specifically, patients older than 80 years faced a higher 60-day mortality and reduced time alive and free from the ICU or mechanical ventilation.^[34]^ Their findings underscore that the physiological burden of aging alone can create different prognoses. In our study, case 4 was an elderly patient with age-related physiological impairments, including reduced pulmonary compliance, impaired immune responses, and diminished physiological reserve, which exacerbated lung injury and hindered recovery. This suggests that the therapeutic efficacy of colistin is not solely determined by achieving target plasma concentrations, but is also critically dependent on the patient’s underlying physiological state and resilience.

AKI developed in cases 2 to 5, with the etiology appearing multifactorial and case-specific. While sepsis is a well-established cause of AKI, it was only present in 2 patients (cases 3 and 4). The onset of sepsis in case 3 occurred after the initial AKI, suggesting that sepsis acted as an aggravating factor for the progression of AKI to stage 2 rather than the primary cause. For case 2, who had normal renal function and received no other nephrotoxins, the most plausible explanation for the AKI is CMS therapy. In contrast, the severely impaired baseline renal function of cases 4 and 5 was likely a major predisposing risk factor that converged with CMS nephrotoxicity. Therefore, in our study, CMS therapy and preexisting renal function were the predominant drivers of AKI, with sepsis serving as a compounding contributor in case 3.

ARC, characterized by increased renal filtration (i.e., CrCl > 130 mL/min/1.73 m^2^), often occurs in young male patients.^[35,36]^ Underlying diseases such as trauma, surgery, sepsis, burns, subarachnoid hemorrhage, and hematological malignancy are risk factors for ARC.^[36]^ Case 1 had ARC with a baseline CrCl of 149.56 mL/min/1.73 m^2^ and received CMS at 9 MIU daily. His blood culture remained positive for CRKP at discharge. This outcome had 2 likely causes. First, the *f*AUC/MIC failed to reach the higher target required for pulmonary infection (*f*AUC/MIC > 48). Additionally, the attainment of colistin therapeutic concentrations was delayed due to the inherent time required for CMS to convert to its active form.^[13,37]^ This delay is more evident in patients with ARC, as enhanced renal function accelerates the clearance of CMS, resulting in reduced conversion to colistin. The 2019 international consensus guidelines recommended a loading dose of 9 MIU followed by the first maintenance dose 12 to 24 hours later.^[38]^ Plachouras et al administered a loading dose of 9 to 12 MIU of CMS, followed by a maintenance dose of 4.5 MIU of CMS every 12 hours, to their critically ill patients.^[39]^ Moreover, at high concentrations, colistin is associated with a moderate post-antibiotic effect and a high unbound colistin fraction,^[38,39]^ which may increase antimicrobial treatment efficiency. However, it is important to note that an inappropriate loading dose can increase the risk of nephrotoxicity. In general, the benefits of an appropriate loading dose outweigh the potential nephrotoxicity risk; therefore, TDM may serve as a useful tool to balance this risk-benefit ratio. Regrettably, none of our patients received a loading dose, which precluded the use of TDM for such an analysis in this study. It simultaneously highlights a promising direction for future research.

## 6. Conclusion

In conclusion, TDM may aid in achieving relatively high but less toxic plasma colistin concentrations, balancing the nephrotoxicity risks with efficacy. However, it should be noted that achieving PK/PD targets is not the only factor influencing clinical outcomes. Due to the nature of our observational study and the small sample size, interventional or controlled studies with larger cohorts are needed in the future to validate these observational findings. Nevertheless, the TDM of colistin is being increasingly implemented at the laboratory level, indicating its importance in assisting clinicians with dose optimization. Additionally, the LC-TQ-MS incorporating MSPE as a sample pretreatment merits wider application in the TDM of colistin.

## Acknowledgments

We extend our sincere gratitude to the patients for their valuable contributions. We also thank the healthcare workers at Tongde Hospital of Zhejiang Province for their collaborative efforts.

## Author contributions

**Conceptualization:** Juan Wang.

**Data curation:** Qiuyu Zhu.

**Methodology:** Xiaoting Gong.

**Investigation:** Qiuyu Zhu.

**Project administration:** Xufen Xia, Yuexing Tu.

**Resources:** Fangqiong Li.

**Supervision:** Juan Wang.

**Validation:** Ji Shen.

**Visualization:** Bo Yang.

**Writing – original draft:** Qiuyu Zhu, Juan Wang.

**Writing – review & editing:** Xufen Xia, Yuexing Tu.
